# Evaluating the safety of orexin receptor antagonists on reproductive health and sexual function

**DOI:** 10.1038/s41380-024-02858-1

**Published:** 2024-11-28

**Authors:** Kenji Hashimoto

**Affiliations:** Chiba University Center for Forensic Mental Health, Chiba, 260-8670 Japan

**Keywords:** Depression, Diagnostic markers

Insomnia is a common sleep disorder affecting people worldwide, characterized by difficulty falling asleep, staying asleep, or waking up too early. It can lead to significant health, economic, and social consequences, including an increased risk of cardiovascular disease, depression, and impaired daily functioning. Benzodiazepines have been widely used to treat insomnia due to their sedative effects; however, there are concerns about dependency, tolerance, and other side effects. New orexin receptor antagonists, which target the orexin system involved in wakefulness regulation, are emerging as a promising treatment for insomnia. These newer medications are considered to have a better safety profile, with a lower risk of dependency and fewer cognitive side effects compared to benzodiazepines [1].

**Fig. 1:**
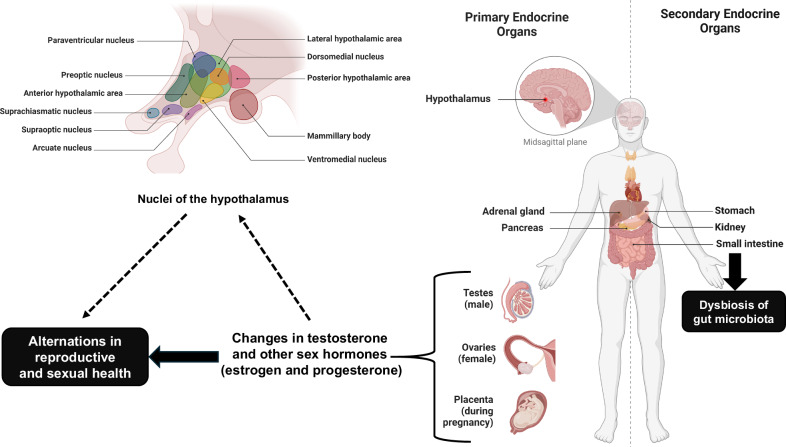
Potential effects of orexin receptor antagonists on reproductive and sexual health. This figure illustrates the distribution of orexins and their receptors (OX1R and OX2R) in the human body. The right side highlights the primary and secondary endocrine organs where orexin receptors are present, including the hypothalamus, gastrointestinal (GI) tract, and reproductive organs such as the testes (in males), ovaries (in females), and the placenta (during pregnancy). The left side shows the nuclei of the hypothalamus in the human brain, where orexins are primarily active. The use of orexin receptor antagonists may affect gut microbiota composition, potentially leading to dysbiosis. Blocking orexin receptors with these antagonists could influence levels of testosterone and other sex hormones (estrogen and progesterone), impacting brain function and, consequently, reproductive and sexual health. Given the important roles of orexins and their receptors in reproductive organs, chronic use of orexin receptor antagonists may have implications for reproductive and sexual health. This illustration was created using resources from BioRender.com, with permission obtained.

Orexins, also known as hypocretins, are neuropeptides that play a crucial role in regulating wakefulness and arousal. Discovered in the rat hypothalamus by Sakurai and colleagues in 1998 [2], moderate amounts are also found in the testis. There are two types of orexins: orexin-A and orexin-B, which bind to two receptors, orexin receptor 1 (OX1R) and orexin receptor 2 (OX2R). These receptors are widely distributed throughout the brain, including areas involved in wakefulness, such as the hypothalamus (Fig. 1) [3]. Several dual OX1R and OX2R antagonists, such as suvorexant, lemborexant, and daridorexant, are widely used to treat insomnia. These antagonists do not have the side effects associated with benzodiazepines [1].

In addition to their presence in the brain, orexins and their receptors are expressed in various peripheral tissues, where they perform diverse physiological functions. They are localized in the gastrointestinal (GI) tract, including the stomach, small intestine, and colon [4], as well as in the reproductive organs such as the testis, ovaries, and uterus [5–7] (Fig. 1). According to the Human Protein Atlas Database (https://www.proteinatlas.org/), the protein expression of OX1R is highest in the testis among all organs. Their distribution in these peripheral tissues suggests that orexins may have broader roles beyond the central nervous system, influencing various aspects of metabolism, digestion, and reproductive health.

Nakabayashi et al. [4] found that orexin-A immunoreactivity was present in various locations, including ganglion cells of the thoracic sympathetic trunk, myenteric plexuses, endocrine cells within the GI tract, pancreatic islet cells, and both syncytiotrophoblasts and decidual cells of the placenta. Furthermore, mRNA expression of prepro-orexin was observed in multiple tissues, such as the kidney, adrenal gland, pancreas, placenta, stomach, ileum, colon, and colorectal epithelial cells [4]. The presence of orexins and their receptors in the GI tract suggests their involvement in key physiological processes, including GI motility, regulation of gastric acid secretion, appetite and feeding behavior, modulation of the enteric nervous system, and impact on gut microbiota [8]. Considering the potential role of the gut microbiota in the immune system [9], it is plausible that orexin receptor antagonists could alter the composition of gut microbiota, affecting immune system function and reproductive health (Fig. 1). Diarrhea, a GI side effect of these antagonists, may result from dysbiosis following their administration. Further studies are needed to explore gut microbiota composition and microbes-derived metabolites in patients with insomnia treated with orexin receptor antagonists.

Orexins and their receptors are also found in reproductive organs (Fig. 1). Administering a bilateral intratesticular injection of the OX1R antagonist SB-334867 at doses of 4 and 12 μg/mouse resulted in reduced testis weights, degenerative alterations in the seminiferous tubules, and changes in steroidogenesis, evidenced by decreased testosterone and increased in 17β-estradiol levels in both serum and in testis [6]. Additionally, treatment with the antagonist led to downregulation of 17β-hydroxysteroid dehydrogenase (HSD) and upregulation of 3β-HSD expression in the testis [6]. This study indicated that OX1R antagonist treatment caused significant reductions in testis weight and degenerative changes in the seminiferous tubules. The notable decrease in the diameter of the seminiferous tubules and the height of the germinal epithelium in treated mice suggests a detrimental impact on spermatogenesis. Given the role of 17β-HSD and 3β-HSD in steroidogenesis in the testis, alterations in these enzymes by the OX1R antagonist could lead to changes in testosterone and other androgens **(**Fig. 1). Moreover, a single systemic dose of SB-334867 (20 mg/kg) reduced certain male sexual behaviors in adult rats, including longer intromission latency and lower ejaculation frequency [10]. This suggests that orexin receptor antagonists may influence male sexual behaviors.

Additionally, the immunolocalization of orexin-B and OX2R in the seminiferous tubules, especially in leptotene, pachytene spermatocytes, round and elongating spermatids, as well as in Leydig cells and Sertoli cells, suggesting a possible regulatory role in spermatogenesis and steroidogenesis [7]. A recent study revealed that the knockdown of OX2R in adult mouse testis potentiated testosterone production [11]. The presence of these receptors in Sertoli cells, which support and nourish developing sperm cells, indicates that orexin signaling could influence sperm maturation and development [6]. Given the role of these androgens in male reproductive development, function, and overall endocrine health, these findings suggest a potential role of orexins and their receptors in regulating steroidogenesis and spermatogenesis in the testis of adult mice [6]. Therefore, it is plausible that orexin receptor antagonists may influence male reproductive health and endocrine function.

Orexins and their receptors, OX1R and OX2R, also play a role in the ovaries, influencing various aspects of ovarian function and reproductive health (Fig. 1) [12]. A study showed increased ovarian expression of both OX1R and OX2R during the proestrous afternoon, dependent on hormones but not the dark-light cycle [12]. Orexin receptor antagonists, such as the OX1R antagonist SB-334867 and the OX2R antagonist JNJ-10397049, reduced proestrous gonadotropins and/or ova numbers while causing ovarian structural changes [12]. In 2013, Yilmaz et al. [13] reported that serum levels of orexin-A were lower in women with polycystic ovary syndrome (PCOS) compared to healthy subjects. A new study using a PCOS model in rats demonstrated that a combination of the OX1R antagonist SB-334,867 and the OX2R antagonist JNJ-10,397,049 significantly reversed elevated serum testosterone levels in the PCOS rats [14]. Collectively, orexins and their receptors in the ovaries are involved in critical processes related to follicle development, hormone production, ovulation, and overall ovarian function. Understanding these roles may offer new avenues for research and treatment in reproductive health and fertility.

The orexin system in premenopausal and postmenopausal women is influenced by hormonal changes, particularly the fluctuations in estrogen and progesterone that occur during menopause [15]. In premenopausal women, these fluctuating hormone levels help regulate the orexin system, balancing arousal and sleep. In postmenopausal women, the significant drop in estrogen and progesterone can disrupt orexin signaling, leading to increased sleep disturbances and other physiological changes (Fig. 1). Understanding this interaction is crucial when considering treatments like orexin receptor antagonists, especially for sleep disorders in peri- and post-menopausal women.

While these medications are effective for improving sleep, their impact on other physiological functions of orexins, including reproductive health, requires careful consideration. Altered orexin signaling may influence hormone levels such as testosterone, estrogen, and progesterone, potentially leading to reduced testosterone in men and menstrual irregularities in women, which could result in decreased sexual desire (Fig. 1). Much of the research discussed in this article has been conducted in animal models, and direct clinical evidence in humans is still limited. Additionally, since the orexin system in the mesolimbic pathways regulates reward and motivation, it is important to monitor for any mood changes, such as depression, in insomnia patients treated with orexin receptor antagonists.

In conclusion, sexual dysfunction caused by selective serotonin reuptake inhibitors is often underestimated due to underreporting by patients, under-recognition by healthcare providers, and the overlap with depression-related symptoms. Many patients may feel uncomfortable discussing sexual health issues with their healthcare provider due to stigma or embarrassment. Therefore, healthcare providers should carefully consider these potential risks when prescribing orexin receptor antagonists, particularly for individuals with reproductive health concerns or sexual function issues. Future studies are needed to explore the long-term effects of these antagonists on reproductive and sexual health in people with insomnia.
